# High-risk HPV prevalence and genotype distribution among women in Liaocheng, Shandong Province, China from 2016 to 2022

**DOI:** 10.3389/fpubh.2023.1145396

**Published:** 2023-03-30

**Authors:** Li-li Zheng, Shuang-feng Chen, Fei Yang, Wei-hua Wang, Cong Xu, Li-yuan Zheng

**Affiliations:** ^1^Central Laboratory of Liaocheng Peoples' Hospital, Liaocheng, Shandong, China; ^2^Department of Clinical Laboratory Liaocheng City Dongchangfu District Maternal and Child Health Hospital, Liaocheng, Shandong, China; ^3^State Key Laboratory of Microbial Technology, and Marine Biotechnology Research Center, Shandong University, Qingdao, China

**Keywords:** human papillomavirus, prevalence, genotype, cervical cancer, vaccine

## Abstract

Human papilloma virus (HPV) infection and its associated disease are major problems affecting millions of individuals around the world. The distribution of HPV genotypes is specific to different areas and different populations. Therefore, understanding the prevalence and genotype distribution of HPV in different populations in different geographical regions is essential to optimize HPV vaccination strategies and to maximize vaccine effects. In this study, 34,076 women from January 2016 to July 2022 were retrospectively analyzed at Liaocheng People's Hospital. Of these, 7540 women were high-risk HPV positive and the infection rate was 22.13%. The top ten genotypes were as follows in descending order: HPV16, HPV52, HPV58, HPV53, HPV39, HPV59, HPV66, HPV51, HPV18, and HPV56 and the least frequent genotypes were, in order, HPV 26, HPV45, and HPV82. The HPV16 positive infection rate was 25.37% and was reduced with the increase in the number of individuals who had undergone HPV screening. The HPV52 infection rate increased with increasing numbers of individuals undergoing HPV screening, and then remained unchanged. The proportion of 20–29-year-olds among all positive women began to decrease since the vaccine was available in 2018. The 30–39-year-old group accounted for the highest percentage of positive women, and the 50–59-year-old group of HPV-positive women with cervical cancer accounted for most infections. This study confirmed that HPV16, HPV52, HPV 58, and HPV53 is widely distributed in this population and the total HR-HPV infection rate remains high in this region. Our findings indicate that prevention of HPV infection in this region still faces important challenges.

## Introduction

Human papilloma virus (HPV) infection and its related diseases remain a major public health concern throughout the world, with 690,000 cases of HPV-induced cancer in 2018, including 604,127 cases of cervical cancer (CC) and 341,831 deaths (1). HPV infection places an enormous burden on both individuals and the health care system. Cervical cancer caused by HPV is the fourth most common cancer among women worldwide (2). In 2020 alone, China recorded ~109,741 new cases of cervical cancer (18.2%) and 60,590 deaths (17.3%) (3).

HPV has many genotypes, and its infection is widespread in the population (4). Until now, ~450 HPV genotypes have been isolated and sequenced (5). HPV genotypes are clinically classified based on the *L*1 gene sequence alignment and their oncogenic risk by the World Health Organization (WHO). The most frequent high-risk HPV (HR-HPV) genotypes of cancer include HPV16, 18, 31, 33, 35, 39, 45, 51, 52, 56, 58, and 59; HPV68 is the second most frequent genotype associated with potential carcinogenicity (2a), and several other HPV types (26, 53, 66, 67, 70, 73, 82, 30, 34, 69, 85, and 97) are also considered HR-HPV and are thought to be potentially carcinogenic (2b) (6).

Persistent HR-HPV infection is the main pathogenic factor (7). HR-HPV plays a key role in both cervical invasive squamous cell carcinoma (SCC) and most cervical adenocarcinoma (EAC) lesions (8–13). Viral oncogenes play the pivotal role in the occurrence of these pathological processes, such as E5–E7. E5 is known to play an important role in early cervical carcinogenesis by upregulate VEGF through EGFR, MEK/ERK1 and 2 along with PI3K/Akt, which induces cell invasion and metastasis (14, 15), while E6 and E7 are the key genes in driving the cells toward oncogenesis (16, 17). E6 protein binds to the cellular tumor suppressor protein p53 and induces degradation of p53 protein (18). E7 protein forms a complex with retinoblastoma protein (pRb) and degrades pRb through the ubiquitin-proteasome pathway (19, 20). The development of HPV-mediated cervical carcinogenesis is a multistep process, and cervical intraepithelial neoplasia (CIN) is a cervical precancerous lesion. In November 2020, the WHO announced a global strategy to accelerate cervical cancer elimination, setting HPV vaccination coverage to 90% by 2030, screening coverage of 70% for cervical lesions, and accessibility to cervical cancer treatment of 90% (21). Together with the European Beating Cancer Plan (22), this approach has greatly facilitated the elimination of all cancer causes by HPV. Cervical cancer is expected to be the first cancer that can be prevented and eliminated. However, the prevalence of common HPV genotypes varies in different regions and across different populations (23, 24); understanding the ecological diversity of HPV prevalence and genotype distribution between various populations in different geographical regions and at different times is essential for optimizing HPV vaccination strategies and to maximize vaccination effects.

The city of Liaocheng is located on the border of three provinces with a dense population and has a relatively poorly advanced economy in Shandong province. The Liaocheng People's Hospital is a typical regional medical center in Shandong Province and is well-known in the border area of Hebei-Shandong-Henan Province. Thus, HPV prevalence and genotype distribution in this region have important significance for Chinese epidemiological data and can provide an important rationale or reference for Chinese public health measures on cervical cancer prevention.

## Materials and methods

### Data sources

In this study, the data of 34,076 female from the Liaocheng People's Hospital between January 2016 and July 2022 were retrospectively analyzed. Samples were collected using a sterile disposable cervical sampler and stored in a cell preservation solution (Tellgen, China). The HR-HPV positive cases were 7,540, of which 3,943 women aged 16–87 years were voluntary simultaneously screened for cervical cytology. The studies were reviewed and approved by Medical Ethics Committee of Liaocheng People's Hospital.

HPV positivity was identified by PCR, and the HPV PCR-Flow fluorescence assay was used for HPV genotyping. Seventeen HR-HPV genotypes were tested using the Nucleic Acid genotyping Kit for Human Papillomavirus (Tellgen, China), including those closely related to cervical cancer (HPV16, 18, 31, 33, 35, 39, 45, 51, 52, 56, 58, 59, 66) and those associated with cervical cancer (HPV26, 53, 68, 82).

### Statistical analysis

The prevalence of HPV infection, HPV genotypes of different grade, and the presence of single or multiple HPV infections, as well as their corresponding 95% confidence intervals (CIs), were estimated using binomial distribution analysis. The chi-square test was performed to compare differences in infection rates over time. A *P*-value < 0.05 was considered statistically significant. Statistical analysis was performed using SPSS version 17.0 (SPSS, IBM, USA).

## Results and discussion

### HR-HPV prevalence among 34,076 women

**Figure 1:**
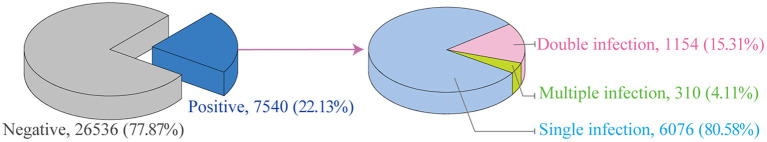
High-risk human papilloma virus (HR-HPV) infection rates in 34,076 cases of women.

Between January 2016 and July 2022, the HR-HPV infection rates in 34,076 women were determined (Figure 1). A total of 7,540 HR-HPV positive cases were identified, and the total infection rate of 17 genotypes was 22.13% (7,540/34,076, 95% CI 21.69–22.57). The HR-HPV positive rate in this study was higher than the overall prevalence of HR-HPV positive (19.1%, range 15.3–24.4%) in China from July 2018 to June 2019 (25) and in Latin America (16%) in 2012 (23), which were similar to those in rural North China (22.2%) in 2017 (26) and in sub-Saharan Africa (24%) and in eastern Europe (21%) in 2012.

HPV DNA screening showed that single genotype infections were significantly more prominent than double (15.30%) or multiple infections (4.11%) (Figure 1). In this study, the infection rate for single genotype infections was 80.58%, which was comparable to those reported in Sichuan (77.2%) in November 2015 and August 2021 (27). The multiple infections in this study were similar to those observed in Yunnan Province (4.2%) (28, 29), but were lower than those reported in atypical squamous cells of women of undefined significance in Sichuan (22.8%) (27). The high rate of multiple infections may be associated with higher incidence of degree of cervical lesions, which was in agreements with the report that multiple infections could increase the risk of cervical cancer (30). Therefore, HPV screening is crucial for all women in this region.

### Genotype distribution in 7,540 women

**Figure 2:**
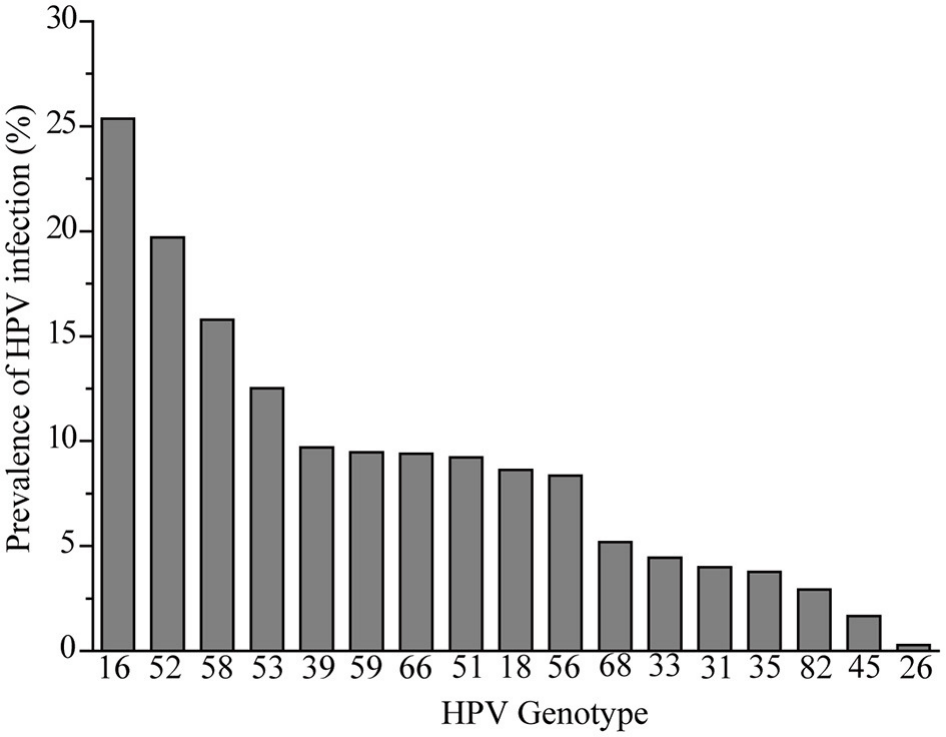
Genotype distribution among 7,540 women.

The infection rate of common HR-HPV genotypes was retrospectively analyzed. Seventeen different HPV genotypes were identified among the 7,540 HR-HPV positive women. As shown in Figure 2, the most common genotype was HPV16, and the top ten most prevalent genotypes were as follows in descending order: HPV16 (25.37%), HPV52 (19.71%), HPV58 (15.78%), HPV53 (12.52%), HPV39 (9.71%), HPV59 (9.46%), HPV66 (9.40), HPV51 (9.23%), HPV18 (8.63%), and HPV56 (8.36%); the least prevalent genotypes in order were HPV 26 (0.28%) and HPV45 (1.67%).

The prevalence of HPV prevalence in women around the world had significant regional variations. In western countries or in continents of Europe/ America, such as Guatemala, HPV18 is the second most prevalent (31, 32). However, in west Africa Lomé, Togo, the most common genotypes are HPV56 and HPV51 (33). In Asian countries, HPV58 and HPV52 are becoming more prevalent than HPV18 (34, 35). The HR-HPV genotypes detected the most frequently were HPV52, HPV16, HPV53, HPV58, HPV51, and HPV68 in China (26). Similar with our study, the top HPV genotype was HPV16 in Hengyang and Shanxi, while HPV58 was the second most prevalent in Hengyang (36, 37). Thus, popular HPV types are not identical in different countries or in different areas of China. The differences in prevalence of HPV in Liaocheng populations may be the women investigated in this study were women with gynecological diseases, of different ethnicity, lifestyle, and urbanization.

### HPV prevalence of vaccine-targeted genotypes in 7 years

The currently approved 9-valent HPV vaccine has the highest valency of all vaccine types, targeting HR-HPV16, 18, 31, 33, 45, 52, and 58. As shown in Table 1, the prevalence of genotypes targeted by the HR-HPV16 vaccine (5.61%), HPV52 (4.36%), HPV58 (3.49%), and HPV 18 (1.91%) was higher in this study. Other vaccine-targeted genotypes HPV45 (0.37%), HPV31 (0.88%), and HPV33 (0.98%) were rarely identified in women (Table 1), with positivity rates <10%. However, the prevalence of genotypes HR-HPV53 (2.77%), HPV39 (2.15%), HPV59 (2.09%), HPV 66 (2.08%), HPV51 (2.04%), HPV56 (1.85%), and HPV 68 (1.15%), which were genotypes not targeted by the effective vaccine, was also high in this study.

**Table 1:** Prevalence and genotype distribution among 34,076 women.

**HPV genotype**	**Positive**	**Total test**	**Ratio in all (%)**
16	1,913	34,076	5.61
18	651	34,076	1.91
31	301	34,076	0.88
33	335	34,076	0.98
35	284	34,076	0.83
39	732	34,076	2.15
45	126	34,076	0.37
51	696	34,076	2.04
52	1,486	34,076	4.36
56	630	34,076	1.85
58	1,190	34,076	3.49
59	713	34,076	2.09
66	709	34,076	2.08
26	21	34,076	0.06
53	944	34,076	2.77
68	391	34,076	1.15
82	221	34,076	0.65
Total	7,540	34,076	22.13

**Figure 3:**
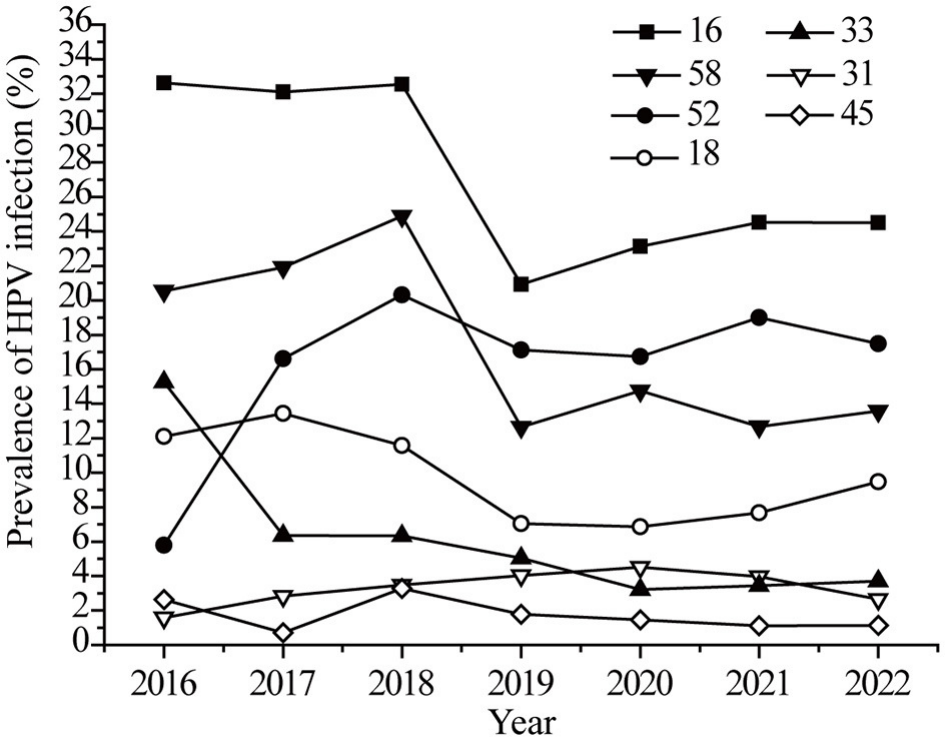
The vaccines HPV prevalence in 7 years.

The infection rates of targeted vaccine genotypes in the last seven years was analyzed. As shown in Figure 3, the HPV16 infection rate showed a sudden drop (32.53 to 20.92%) in 2019, and then the declining rate slowed. The changing trend for HPV 18 and 58 were similar to that of HPV 16. The HPV52 infection rate showed an obvious growth trend (5.79 to 19.34%) from 2016 to 2017, and then leveled off (Figure 3). There were no significant changes in the HPV 32, 33, and 45 infection rates. It should be noted that HPV screening was added to female medical physical examination protocols in 2019, with more healthy women involved in screening. Therefore, the trends observed for HPV 16, 18, and 58 were possibly not only due to the effects of the vaccine but also to the increased screening.

**Figure 4:**
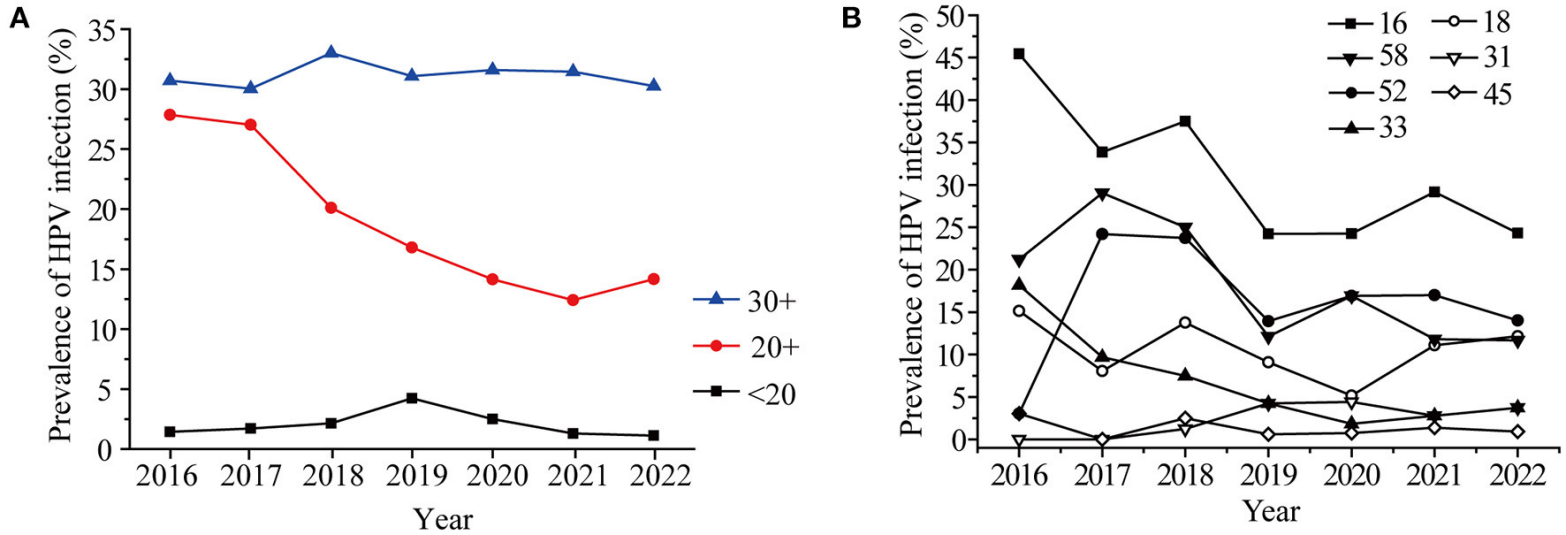
Prevalence of HR-HPV in women under 40 years old **(A)** and Prevalence of seven HR-HPV genotypes in women aged 20-29 years old in different years **(B)**.

The percent of vaccine genotypes in different age groups was further analyzed, as shown in Figure 4A, the proportion of vaccine genotypes in 20–29-year-old females markedly decreased from 27.86% in 2016 to 12.41% in 2021 (*P* = 0.01) (Table 2). The proportion of vaccine genotypes in the 15–20-year-old women increased in 2018, peaked in 2019 (4.23%), and then declined (*P* = 0.56), while no obvious changes were observed in the 30+ year-old groups (*P* = 0.92) (Figure 4A, Table 2). The HPV vaccine has been available in China since 2018, which led to increased awareness of HPV screening among young people, and the change in HPV infection in individuals aged 20–29 years may be due to the efficacy of the vaccines. Furthermore, the prevalence of HPV genotypes targeted by vaccines was different in individuals aged 20–29 years (Figure 4B). The ratio in positivity of HPV16 in 20–29-year-olds decreased slightly from 2018 to 2022, but the change was not significant (*P* = 0.88).

**Table 2:** Annual age distribution in 7,540 HR-HPV positive women.

	**2022**		**2021**		**2020**		**2019**		**2018**		**2017**		**2016**		**Total**	
**Age**	***n***	**%**	***n***	**%**	***n***	**%**	***n***	**%**	***n***	**%**	***n***	**%**	***n***	**%**	***n***	**%**
15–20	17	1.13	30	1.29	48	2.49	42	4.23	9	2.15	4	1.72	2	1.43	152	2.02
20+	214	14.17	288	12.41	272	14.14	167	16.80	84	20.10	63	27.04	39	27.86	1,127	14.95
30+	457	30.26	730	31.45	608	31.60	309	31.09	138	33.01	70	30.04	43	30.71	2,355	31.23
40+	350	23.18	611	26.32	535	27.81	263	26.46	106	25.36	53	22.75	30	21.43	1,948	25.84
50+	352	23.31	510	21.97	335	17.41	157	15.79	46	11.00	32	13.73	18	12.86	1,450	19.23
60–87	120	7.95	152	6.55	126	6.55	56	5.63	35	8.37	11	4.72	8	5.71	508	6.74
Total	1,510		2,321		1,924		994		418		233		140		7,540	

*N*, numbers; %, Proportion of different age groups in the HR-HPV positive women.

Different vaccines achieve cross-protection to potential precancerous lesions, in the case of effective vaccines, a decline in the incidence of global CIN 3 of 25.8% has been achieved in 6 years (38). In Denmark, Norway, and Sweden, the HPV infection rate decreased from 36.5% in 2006–2008 to 34.5% in 2012–2013 after the vaccine was introduced (39). In early 2018, the vaccine was already available in China. The HPV16, 18, and 58 infection rates showed a slow drop after 2019 in this study, and the vaccine may play important roles. However, the other vaccine genotypes except for HPV16 did not achieved a reduction in prevalence among 20–29-years-old individuals in our study. Furthermore, the prevalence of HR-HPV, especially HPV52 and HPV58, is higher in black rural women (40). HPV52 (21.7%) is the most common HR-HPV genotype in rural North China (26). The increasing prevalence of HPV52 from 2016–2017 in this study may be associated with rural women. Therefore, the HPV vaccine promotes the public's understanding of HPV, although it is not yet very common in this region. Meanwhile, it is worth noting that HPV 53, 39, 59, 66, 51, 56 were also the most commonly observed in the present study. However, they are still not covered in the latest 9-valent HPV vaccine, these HR-HPV types should attract the attention of HPV vaccines researchers.

### Distribution of cervical lesions in 3943 HR-HPV positive women

**Figure 5:**
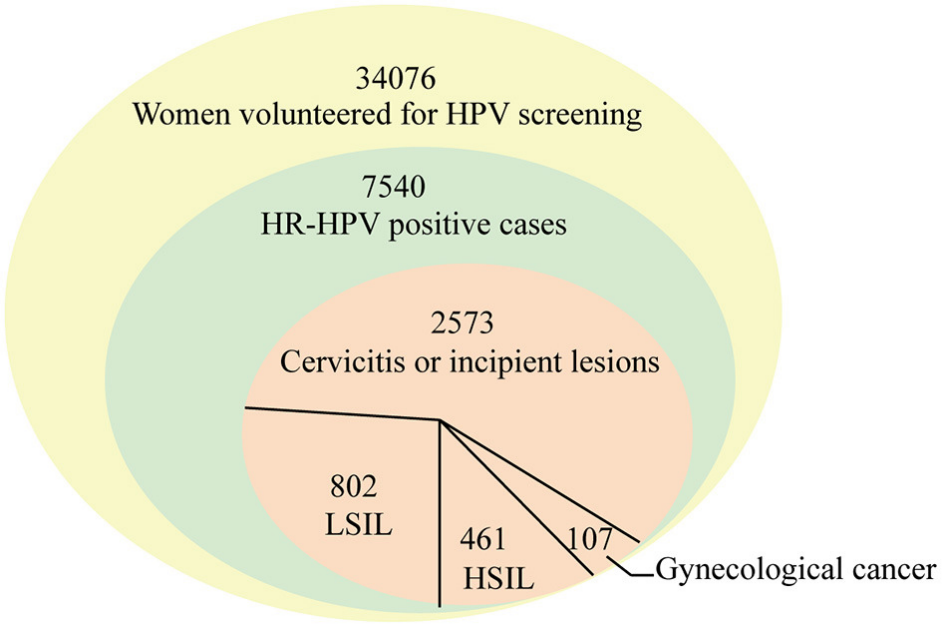
The number of participants in each procures.

Among 3,943 women receiving cervical cytology examination, as seen in Table 3 and Figure 5, women with cervicitis or incipient lesions were 2,573 (65.25%), women with low-grade squamous intraepithelial lesion (LISL) were 802 (20.34%), and women with high-grade squamous intraepithelial lesion (HISL) were 461 (11.69%). Furthermore, 107 cases (2.71%) of gynecological cancer were identified, including cervical squamous cell carcinomas (2.16%), adenocarcinoma (0.25%), endometrial cancer (0.25%), teratoma (0.03%), and ovarian cancer (0.03%). The most frequently HR-HPV genotypes were HPV16, HPV58 and HPV52 in women with gynecological cancer (43.97, 22.43, and 16.82%), HISL (46.20, 17.79, and 13.88%), LISL (24.44, 17.21, and 15.46%) and cervicitis or incipient lesions (20.05, 18.77, and 13.76%). HPV16 was the highest HR-HPV genotype in women with gynecological cancer and HISL compared with other two groups (*P* = 8.5E-05), while no obvious changes of other two genotypes HPV52 and HPV58 were observed in each group (HPV52: *P* = 0.90; HPV58: *P* = 0.97). It can be seen that HPV16 plays a key role in cervical lesions.

**Table 3:** Proportion of different lesion grades in 3,943 HR-HPV positive women with cervical cytology.

**Lesions grades**	**Numbers**	**Proportion (%)^a^**
Cervicitis or incipient lesions	2,573	65.25
LSIL	802	20.34
HSIL	461	11.69
Gynecological cancer	107	2.71
Cervical squamous cell carcinomas	85	2.16
The cervical adenocarcinoma	10	0.25
Endometrial cancer	10	0.25
Teratoma	1	0.03
Ovarian cancer	1	0.03

a The proportion of Lesions grades were calculated relative to 3,943 HPV positive women with cervical cytology.

HSIL, High-grade squamous intraepithelial lesion (cytology based); LSIL, Low-grade squamous intraepithelial lesion (cytology based).

Our findings were similar to those of previous studies, in which HPV infection was closely associated with cervical squamous carcinoma and adenocarcinoma (9–14). HR-HPV infection may also be associated with the appearance of primary ovarian squamous cell carcinoma (41). The estimated incidence rate of cervical cancer was 6.7–11.2 per 100,000 in 2008 in Central Arkansas (24). The rate of cervical cancer showed a higher incidence (85/34,076) in this study (Tables 1, 3). Furthermore, the prevalence of cytologic cervical pathology is directly proportional to the severity of the lesion. Globally, the HR-HPV positive rate in women with normal cytology was around 11–12%, reaching around 90% in grade 3 CIN or invasive cervical cancer, and 100% in cervical cancer (23). The results suggest that cervical cancer screening is necessary in the local region.

### Age distribution of HR-HPV prevalence

As shown in Table 2, the ages of 7,540 HR-HPV positive women ranged from 15 to 87 years, of which 91.25% (6,880/7,540) were 20–59 years old. HR-HPV infection rates of the women in the 30–39-year group were 31.23% (2,355/7,540) and were the highest (*P* = 0.04), followed by that of the 40–49-year-old group (25.84%, 1,948/7,540) and the 50–59-year group (19.23%, 1,450/7,540). It should be noted that the youngest HR-HPV positive patient was 15 years old, and the youngest cervical cancer patient was 28 years old. A case in young women as young as 19 years old (42) has also been reported. Thus, HR-HPV infection rates of women decreased with increasing age, and the risk of cervical cancer in the younger age groups should also be taken much more seriously.

**Figure 6:**
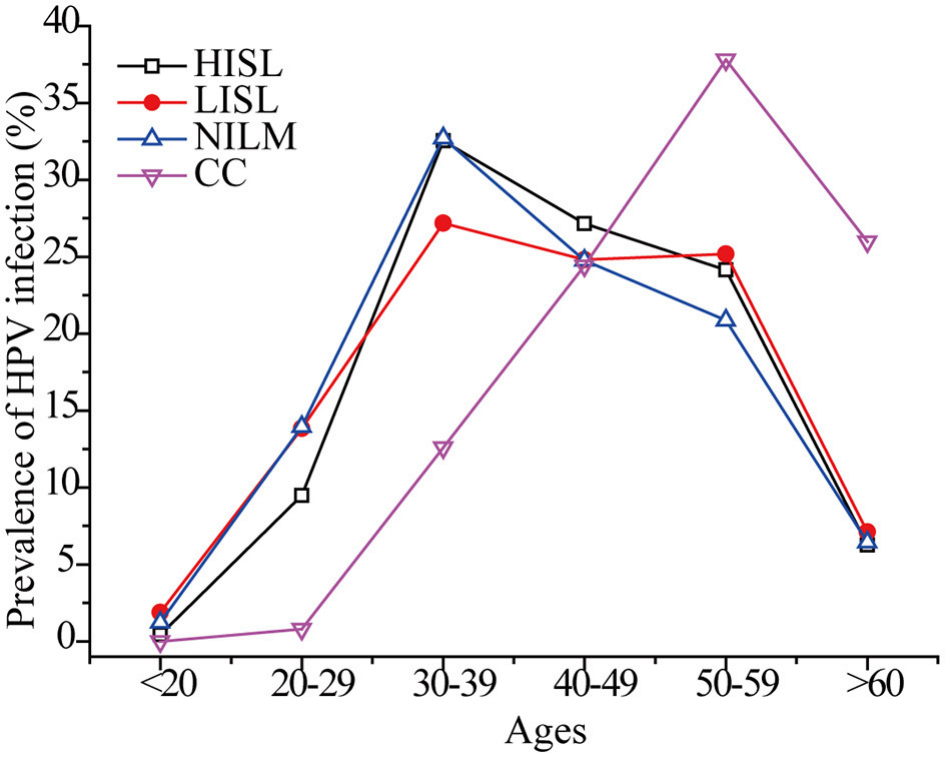
Age distribution of HR-HPV prevalence in 3,943 women.

As shown in Figure 6, the peak incidence of cervical cancer was among 50–59-year-old females. In other age groups, the maximum HR-HPV prevalence rate was observed in women 30–39 years of age, and slowly decreased in older ages, while a second small peak appeared in women 50–59 years of age. The global prevalence of HPV infection in women with normal cytology presents two peaks in women < 25 years and perimenopausal or early menopausal women (3). Similar dual peaks indicating the highest HR-HPV infection rates were observed in women younger than 30 years of age (22.0%) and in women 50 years of age and older (21.8%) (26). The proportion of postmenopausal women with persistent HPV infection is high (43), and middle-aged women may be infected with new HPV genotypes (44). The time from CIN 3 to the progression of invasive cervical cancer is estimated to be 10–20 years or more (45). This may explain the highest infection rates in 30–39 and the highest cervical cancer rates in women aged 50–59 years. Therefore, it is also necessary to detect pathological changes in the genital tract and perform continuous HPV screening in perimenopausal, menopausal, and postmenopausal women.

## Conclusion

This study revealed HPV prevalence and genotype distribution in Liaocheng city, Shandong province, which were mainly consistent with the prevalence of HPV in China. In addition to HPV16, HPV52, and HPV 58, the prevalence of HPV53, HPV39, HPV59, HPV66, HPV51, HPV56, and HPV68 was also high in this region, and this has important guiding role in the optimization and development of vaccine research. This study also indicated the urgent need to improve HPV prevention and control measures.

## Data availability statement

The raw data supporting the conclusions of this article will be made available by the authors, without undue reservation.

## Ethics statement

The studies involving human participants were reviewed and approved by Ethical Review Committees of Liaocheng People's Hospital. The patients/participants provided their written informed consent to participate in this study.

## Author contributions

LZ analyzed and interpreted the patient data and was a major contributor in writing the manuscript. LZ also analyzed and interpreted the patient data collaboratively. CX, SC, FY, and WW collected data. All authors read and approved the final manuscript.
